# Molecularly imprinted fluorescence sensor chip for lactate measurement

**DOI:** 10.1038/s41378-024-00803-4

**Published:** 2024-11-25

**Authors:** Muersha Wusiman, Fariborz Taghipour

**Affiliations:** Chemical and Biological Engineering, University of British Columbia, Vancouver, Canada

**Keywords:** Nanoparticles, Optical sensors

## Abstract

Lactate measurements provide an opportunity to conveniently evaluate bodily functions and sports performance. A molecularly imprinted fluorescence biochip provides an innovative way to achieve lactate measurement and overcomes the limitations of enzyme-based sensors. To realize this goal, ZnO quantum dots (QDs), a biocompatible sensing material, were combined with selective receptors comprised of molecularly imprinted polymers (MIPs). The lactate-selective imprinted polymers were formed using 3-aminopropyltriethoxysilane (APTES) and 5-indolyl boronic acid monomers. Furthermore, a new solid-phase sensing platform that overcomes the limitations of liquid-based sensors was developed to detect lactate in real-time. The platform consists of the biosensor chip with a thin-film sensing layer, an ultraviolet (UV) excitation source, and a portable light detector. The final sensor has a sensitivity of 0.0217 mmol L^-1^ for 0–30 mmol L^-1^ of lactate in phosphate-buffered saline (PBS) with a correlation coefficient of 0.97. The high sensor sensitivity and selectivity demonstrates the applicability of the ZnO QDs and synthetic receptors for sweat analysis.

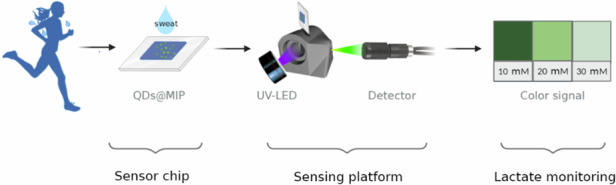

## Introduction

Lactate, the anion form of lactic acid, is a biochemical formed by anaerobic glycolysis, and its concentration is highly relevant for body function^1^. For athletes, the concentration of lactate can indicate their performance by tracking the adenosine triphosphates (ATPs) generating pathway^2,3^. For patients in intensive care, lactate concentration is linked to the possibility of shock, kidney and liver diseases, and heart failure^4–6^. For manufacturers, lactate concentration is used to assess the quality of food products, such as dairy, alcoholic, and non-alcoholic beverages^7^.

Various methods, including high-performance liquid chromatography (HPLC), gas chromatography (GC), electrophoresis, and biosensors, have been used for lactate measurement^8–11^. Among these methods, detection through biosensors is more efficient compared with conventional laboratory equipment and can be used to measure lactate in the blood^12^, sweat^13,14^, and tears^15^. The typical lactate sensors use the enzymatic reaction between lactate and lactate oxidase, which trades electrons during their reaction to generate a sensor response. However, enzymes are costly and immobilizing them causes a significant loss in activity thus impacting the cost and the shelf life of the sensor^13,16^. Therefore, using alternative lactate receptors is the key to obtaining stable sensors with longer shelf life.

Fluorescent sensors are widely used optical sensors that detect targets based on the change in the fluorescence intensity via target quenching. Fluorescent sensors offer high sensitivity, specificity and a straightforward detection method. The sensing mechanisms of fluorescent sensors are based on photo-induced electron transfer (PET) or resonance energy transfer (RET) pathways. PET is triggered when there is a complex formed between the fluorescence molecules and the target molecule that can absorb the excited electrons; RET is induced when there is an alignment between the emission band of the fluorescence molecule and the absorption band of the target molecule. Therefore, fluorescence sensors enable target detection without an intermediate like enzymes. Quantum dots (QDs) are nanometer-sized semiconductors that are commonly used as fluorescence sensing material. Their bright fluorescence and size-tunable emission have attracted great interest in fluorescence sensor development. Due to the toxicity of the Cd-based QDs, green QDs, such as ZnO and carbon QDs, are more suitable candidates for the development of biocompatible fluorescence sensors^17–22^. ZnO QDs have a wide bandgap and a large exciton binding energy which make them one of the brightest QDs^23^. Although ZnO QDs have been extensively used in energy applications, catalysis, and in vitro/in vivo diagnosis, they were rarely used for fluorescence sensor development. Once the challenges of the application and adaptation of ZnO QDs for fluorescence detection in various environments are addressed, they can replace the commonly used toxic and pollution-causing heavy metal-based QDs. In our research, we aimed to leverage the advantages of ZnO QDs, including their non-toxicity, straightforward preparation method, and cost-effectiveness, to explore their potential in fluorescence detection.

To enhance sensor selectivity, molecularly imprinted polymers (MIPs) are often incorporated into fluorescence sensors as the recognition unit^23–25^. MIPs are synthetic polymers formed with the existence of the target molecule and appropriate monomers; the imprinted sites formed during the polymerization match the target molecule in terms of shape, size, and functional group. Therefore, these imprinted sites will selectively bind to the target molecule when re-exposed to the molecule. Compared with biological receptors, such as enzymes, antibodies, and aptamers, MIPs offer comparable target recognizability with higher stability and lower cost. The combination of QDs and MIP forms imprinted fluorescence sensors that feature high sensor sensitivity and selectivity. This type of sensor has been successfully applied for detecting ions^24,26^, chemical contaminants^27,28^, small biomolecules^29,30^, and proteins^31–33^.

The formation of stable and well-defined imprinted sites is the key factor that enables MIPs to show target selectivity, and the quality of imprinted sites is dependent on the interaction between the monomer and the template molecule. Monomers that have been previously reported for imprinting lactic acid can be classified into two types: those with amino-functional groups and those with boronic acid functional groups. Monomers containing amino-functional groups such as allyl amine^8^ and o-phenylenediamine^34^ can form amide bonds with the carboxyl group of lactic acid; monomers containing a boronic acid functional group like 3-aminophenylboronicas^35^ can bind to the *cis*-diol functional group of lactic acid to generate imprinted sites. Although there are previous examples of forming lactic acid imprinted polymers, there is no precedence of combining MIPs with QDs to detect lactic acid, to the best of our knowledge.

Here we present the first study on the development of molecularly imprinted fluorescent sensor chips to measure lactate in sweat. Although molecularly imprinted fluorescence sensing material has been applied as an analytical tool in research labs, the current state of the art is based on liquid samples analyzed using a fluorescence spectrophotometer, which creates for the point-of-care analysis. By applying several fundamental scientific innovations and technological advancements, we transformed this technique from a complex lab-based analysis method to an easy-to-use portable biosensing technique with enhanced sensitivity. The biological compatible ZnO QDs were used as the sensing material and the synthetic receptor MIPs were applied as the recognition material. Imprinted sites were formed using two different monomers to assess their suitability for lactate monitoring: the commonly used monomer 3-aminopropyltriethoxysilane (APTES), which is expected to bind with the carboxyl group of lactic acid, and 5-indolylboronic acid, which is expected to bind with the *cis*-diol group of lactic acid. To realize real-time lactate monitoring, the obtained molecularly imprinted fluorescence sensing material was transformed into a biosensor chip using a facile thin film coating method which allowed signal detection with a simple light/colour detector rather than costly spectrophotometers. This way, the chip will be easy to handle, portable and more sensitive compared with the current state-of-the-art molecularly imprinted fluorescence sensing material, which is based on liquid samples analyzed by fluorescence spectrophotometer. The developed sensors were evaluated for measuring lactic acid in deionized (DI) water first to prove the feasibility of the detection concept and then for measuring lactate in phosphate-buffered saline (PBS), which is a water-based salt solution commonly used in biological research to represent sweat.

## Materials and methods

Sensing materials were formed using ZnO QDs and MIPs. ZnO QDs were synthesized with the sol-gel method, and MIPs were synthesized with the monomers APTES and 5-indolylboronic acid using the reported synthesis method with some modifications. Non-imprinted polymer (NIP)-based sensors were prepared using the same method but without the addition of the template molecule. Several characterization methods were applied to examine the structure, composition, and optical properties of the sensing materials.

### Chemicals and reagents

Zinc acetate dihydrate, lithium hydroxide (LiOH), 3-aminopropyltriethoxysilane (APTES), tetraethyl orthosilicate (TEOS), ammonia solution 2 M in ethanol, 5-indolylboronic acid, hydrogen peroxide (H_2_O_2_), iron (II) chloride, lactic acid, ascorbic acid (AA), uric acid (UA), and glucose were purchased from Sigma-Aldrich (Ontario, Canada).

### Synthesis of APTES functionalized ZnO QDs

ZnO QDs were prepared and functionalized according to the previously reported method^23,36^ with some modifications. First, zinc acetate dihydrate (0.549 g) was added to absolute ethanol (50 mL) and refluxed at 78 °C to obtain a clear solution, and then LiOH (0.2395 g) were added to ethanol (30 mL) and mixed until fully dissolved. The fully dissolved LiOH was added drop-wise to the cooled zinc acetate solution at room temperature under a nitrogen stream, and the solution was stirred for 1 h at 60 °C to complete the reaction. The final turbid solution was centrifuged at 13000 rpm for 20 min to collect the QDs particles. The collected QDs were dispersed in absolute ethanol (20 mL) for functionalization, and then APTES (400 μL) were added drop by drop under continuous stirring, followed by the addition of DI water (300 μL). The mixture was allowed to react by mixing at room temperature for 2 h.

### Synthesis of polymer-capped ZnO QDs

Silica-based imprinted polymer with ZnO QDs embedded in the polymer network during polymerization (ZnO@APTES-MIP) was prepared according to the reported methods^36,37^ with minor modifications. 0.7 g of APTES functionalized QDs were first dispersed in ethanol (20 mL), and then APTES (160 μL) and lactic acid (20 mg) were added to the mixture and stirred for 30 min. Then, TEOS (200 μL) and ammonia solution (200 μL) were added to the mixture (PH = 7) and stirred continuously for 16 h in the dark. The ZnO@APTES-NIP was prepared using the same procedure without the addition of lactic acid. The prepared MIP and NIP were collected and washed with absolute ethanol to remove the template molecules.

Molecularly imprinted poly(indolylboronic acid) (MIP@Pin-BAc) was prepared according to the previously reported method^38^ with some modifications. The 5-indolylboronic acid/lactic acid/ FeCl_2_ (0.1 g/0.1 g/0.05 g) mixture was first dissolved in DI water (50 mL), and the PH of the solution was set to 8 with the addition of NaOH. After the formation of a homogenous solution, H_2_O_2_ (2.5 mL) was added to start the polymerization. No crosslinking agent was used for the polymerization of poly(indolylboronic acid). After 6 h of reaction at room temperature, the polymer was collected and washed with DI water to remove the unreacted monomer and the template molecule. The NIP was prepared using the same procedure without the addition of lactic acid.

To form molecularly imprinted Pin-BAc and ZnO QDs composite (MIP@PIn-BAc/ZnO), centrifuged Pin-BAc (0.26 g) dissolved in DI water (32 mL) and ZnO QDs (0.7 g) dissolved in ethanol (18 mL) were vigorously mixed for 3 h; inspired by a previously reported method^38,39^. The composite was washed with ethanol to remove unattached QDs. The centrifuged product was dissolved in ethanol to prepare the sole for spin coating.

### Solid-phase sensor preparation

The solid sensor was prepared with the physical coating of the sensing material. In specific, the spin coating of the stock solution was completed according to the previously reported technique^40^ with some modifications. A quartz chip measuring 2.5 × 2.5 × 0.15 cm was selected to ensure compatibility with both forward and backward UV illumination. The chip underwent a sandblasting process to optimize deposition. Masking tapes were used to selectively expose only the targeted regions for deposition, creating a 1 cm² square-shaped coating area to regulate distinct active areas. First, the stock solution (200 μL) was added to the quartz chip dropwise while rotating the chip at 500 rpm, and then the speed was increased to 1000 rpm for 30 second. The coated quartz chips were heated for 3 min at 80 °C to fully evaporate the solvent. The procedure was repeated three times to obtain optimum sensing layer thickness.

### Characterization and evaluation methods

Fluorescence measurements of the QDs (with a peak emission around 500 nm) were performed using a Cary Eclipse Fluorescence Spectrometer Agilent Technologies, Inc., California, USA), fluorescence measurements of the solid sensor were performed using a portable spectrometer (USB2000 + ; Ocean Insight, Orlando, USA) with 310 nm UV irradiation (High Power: 2×2 Series; Violumas, California, USA). The morphology was evaluated with high-resolution TEM (Tecnai G20; FEI, Oregan, USA). Absorption properties were recorded using a UV-visible spectrophotometer (Cary 100; Agilent Technology, Inc., California, USA). Fourier transform infrared (FT-IR) spectra were obtained using a Frontier FT-IR Spectrometer with attenuated total reflectance (PerkinElmer Inc., Waltham, MA, USA). The Shared Instrument Facility and Bioimaging Facility at the University of British Columbia provided the characterization instruments.

The performance of the solid sensor was evaluated by measuring the fluorescence intensity before and after the addition of samples (10 μL) in various concentrations. To reduce experimental errors, the solid sensor was placed in a fixed solid sample holder, which allowed 45° of excitation and measurement, respectively, to reduce the interference of UV illumination. Several sets of experiments were performed three times each to obtain error bars, and tables representing a confidence interval (CI) of 95% (equivalent to a significance level of α = 0.05) were generated using Student’s *t*-distribution for the samples.

The selectivity of the sensor was determined by measuring the sensor response to 0.3 mmol L^-1^ glucose, 0.03 mmol L^-1^ AA and 0.05 mmol L^-1^ UA, which are the concentrations of these molecules commonly found in sweat.

## Results and discussion

### Characterization of ZnO QDs based molecularly imprinted fluorescence sensing material

To determine the absorption and the emission band of ZnO QDs, the UV-Vis spectra and the fluorescence spectra of the material were recorded. ZnO QDs absorbed UV radiation between 290 and 340 nm, with a peak value of around 320 nm (Fig. S1A). Upon testing the light emission intensity with various UV excitation wavelengths ranging from 265 to 365 nm, we observed that the emission intensity increased as the UV wavelength approached the absorption peak value. Thus, a commercial UV- light-emitting diode (LED) at a wavelength of 310 nm was used to excite the solid sensing material to allow QDs to generate higher emission intensity. The excited ZnO QDs exhibited symmetric emission spectra, with a peak emission around 500 nm (Fig. S1A) and a blue emission (Fig. S1B).

**Fig. 1:**
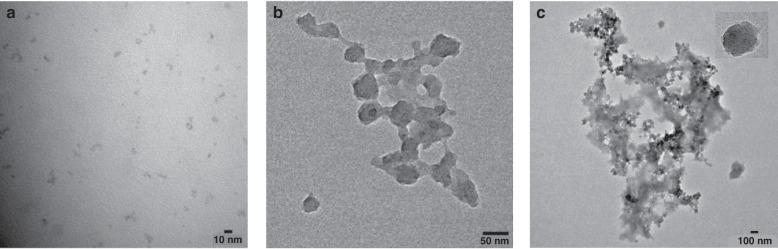
High-resolution images of the sensing material structure. TEM images of ZnO QDs (**a**), MIP@PIn-BAc (**b**), MIP@PIn-BAc/ZnO composite (**c**), and the QDs contained in the polymer (c. inset)

The TEM image of ZnO QDs (Fig. 1a) exhibited that QDs were well dispersed and ranged from 3 to 5 nm in diameter. This was corroborated by calculating the particle size range from the peak emission of the QDs, as depicted in a fitted curve correlating emission peak wavelength with QDs diameter in a previous study^41^. According to this curve, QDs emitting at 500 nm corresponded to an average diameter of approximately 3 nm. If a more precise determination of the average particle size is desired, it can be obtained from the particle size distribution histogram derived from TEM images. However, since the focus of our study is on developing a solid-phase fluorescence sensor, we believe that while knowing the exact particle size of the quantum dots is informative, it is not critical to the sensor development process.

The high-resolution TEM images of MIP@PIn-Bac (Fig. 1b) showed that the PIn-BAc nanoparticles appeared in spherical shapes with a diameter of approximately 30 nm. A similar structure was observed when the ZnO QDs were linked to the MIP@PIn-BAc (Fig. 1c); part of the QDs was at the periphery of the polymer and part of them was inside the polymer, as shown in the inset of Fig. 1c. The observation that QDs residing on the margin of the polymer spheres might be due to the π-π interaction between the PIn-BAc and the sp^2^π clouds of the QDs^42^.

**Fig. 2:**
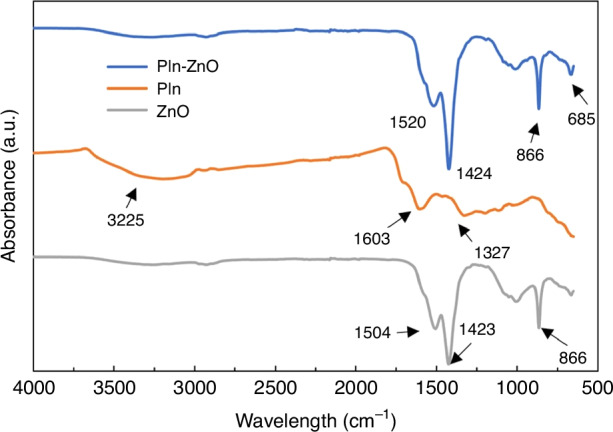
Chemical analysis of the sensing material. FT-IR spectrum of silica ZnO QDs, MIP@PIn-BAc, and MIP@PIn-BAc/ZnO composite

FT-IR spectrums of silica ZnO QDs, MIP@PIn-BAc, and MIP@PIn-BAc/ZnO composite are shown in Fig. 2. The asymmetrical absorption peaks of silica-coated ZnO QDs at 1504 cm^–1^ and 1423 cm^–1^ are due to the stretching of the C-H and O-H groups attached to the QDs from the reactant acetate during the synthesis; the peaks at 866 cm^–1^ and 672 cm^–1^ are assigned to C-H vibration, especially the peak at 672 cm^–1^ is assigned to the C-H group of alcohols that resulted from the repeated washing with ethanol. The Si-O-Si stretching peak that appeared on 1000 cm^–1^ and 1030 cm^–1^ was evidence of growing APTES functional groups on the surface of QDs^43–45^. For the MIP@PIn-BAc, the broad peak from 3625 cm^–1^ to 2990 cm^–1^ is assigned to the N-H stretching, the peak at 1603 cm^–1^ is ascribed to the carbon-carbon double-bound of the cyclic structure, and the peak at 1327 cm^–1^ is attributed to the C-N stretching for aromatic amines^46^. The FT-IR spectrum of the MIP@PIn-BAc/ZnO composite is predominated by the peaks belonging to the QDs. The peaks assigned to C-H, O-H, and Si-O-Si for the QDs can be seen in the composite, thus proving the successful incorporation of the QDs into the MIP structure. X-ray photoelectron spectroscopy (XPS) analysis can be performed alongside FT-IR to investigate surface composition (a few nm in depth) and provide additional information. However, it was deemed unnecessary for this research because the QDs attached to the surface would be deactivated in subsequent stages, thus not contributing to sensor sensitivity. However, the QDs deposited near or within the imprinted sites will remain active and produce a detectable signal.

### Sensor evaluation process

Choosing a suitable monomer to form MIPs enables a strong monomer-template binding which is critical for the selectivity of molecularly imprinted fluorescence sensors. Therefore, monomer optimization should be made for the lactate sensor, especially since this is the first study to apply MIP sensors with QDs as the sensing material for measuring lactic acid, to the best of our knowledge. We evaluated the effects of two different monomers on lactic acid detection; APTES will form the MIP by binding to the carboxyl functional group of the template lactic acid and 5-indolylboronic acid will bind with the *cis*-diol functional group of lactic acid to form the MIP. APTES is commonly used for MIP formation^47–50^, and parametric studies have been conducted for ZnO@APTES-MIP; in contrast, there is very little information on the MIP@PIn-BAc formation and we found no research that reported the incorporation of ZnO QDs to MIP@PIn-BAc. Therefore, efforts were made to determine the optimum synthesis method for MIP@PIn-BAc/ZnO before examining the sensor’s performance on lactic acid detection.

The sensor was first evaluated with lactic acid in DI water to prove its sensing capability; then, to mimic the sweat content, the sensor was further developed to detect lactate in PBS. The fluorescence quenching of both sensors was evaluated using the Stern–Volmer equation^51–53^:

1 $${F}_{0}/F=1+{K}_{{SV}}* {C}_{q}$$

where *F*_0_ and *F* are the fluorescence intensity before and after the addition of the target molecule, respectively, *Cq* is the concentration of the target molecule, and the Stern–Volmer constant *K*_sv_ is the sensitivity of the sensor. The units of fluorescence intensity and the concentrations are arbitrary units (a.u.) and millimolar (mmol L^-1^), respectively.

### Formation of MIP with APTES

Before developing MIP-based sensing materials, we initially studied the response of ZnO QDs to lactate. As depicted in Fig. S2, the QDs exhibited quenching in the presence of lactate, but the correlation of the results was poor. Additionally, we observed erosion on the QDs sensor surface with increasing lactate concentrations. Consequently, we concluded that the addition of a polymer layer to the QDs was necessary to protect the QDs against harsh environments and improve the sensing performance.

The previous APTES-based fluorescence sensor developed by our group^50^ exhibited high sensitivity and selectivity to detect 2,4-D. The superior sensor performance was contributed to the imprinted sites that were formed between the amino functional group of the monomer and the carboxyl functional group of the target molecule. We anticipated that a similar high sensor performance could be obtained for lactic acid using APTES monomer because of the carboxyl functional group of lactic acid. Based on this hypothesis, the lactic acid imprinted polymer was prepared using APTES as the functional monomer and TEOS as the cross-linking agent, following the procedure shown in Fig. S3.

**Fig. 3:**
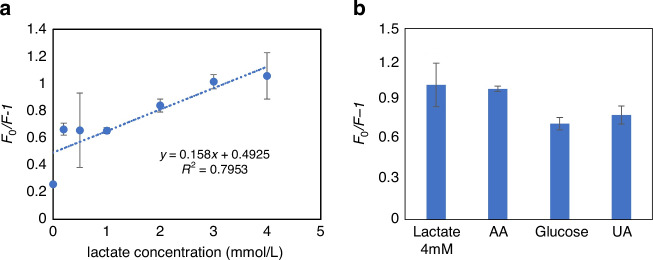
Evaluation of ZnO@APTES-MIP sensor's sensitivity and selectivity. Sensitivity of ZnO@APTES-MIP to lactic acid (**a**) and ZnO@APTES-MIP's selectivity for lactic acid against the interfering molecules (**b**)

The ability of the ZnO@APTES-MIP solid sensor to detect lactic acid was evaluated by recording its response to 0 to 4 mmol L^-1^ of lactic acid. As shown in Fig. 3a, the ZnO@APTES-MIP showed fluorescence quenching proportional to lactic acid concentration and a linear Stern–Volmer relation with a sensor sensitivity of 0.158. To determine the effect of interfering molecules and evaluate the sensor’s capability to selectively measure lactic acid in actual biological samples, several commonly seen interfering molecules in sweat, including glucose, UA, and AA were added to the ZnO@APTES-MIP sensor to record the sensor’s response. The ZnO@APTES-MIP sensor failed to show selectivity toward lactic acid by demonstrating a similar level of response to the interference molecules (Fig. 3b). Since sensor selectivity is an important factor in preventing false-positive response, we concluded that ZnO@APTES-MIP is not applicable for lactate detection.

### Formation of MIP with 5-indolylboronic acid

5-indolylboronic acid contains a boronic acid functional group that is known to have a high binding affinity to compounds with *cis*-diol functional groups such as carbohydrates, glycoproteins, and nucleosides^54,55^. MIP formed by 5-indolylboronic acid was first reported by Fuxiang Chu et al.^38^; the MIPs were formed with the polymerization of the indole group (Fig. S4A), and the selectivity of the MIPs was attributed to the boronic acid side chain that binds with the target molecule (Fig. S4B). The formed polymer has been successfully combined with carbon-based QDs for the fluorescence detection of dopamine^39^ and glucose^38^. Since lactic acid contains a *cis*-diol group similar to dopamine and glucose, 5-indolylboronic acid was used as the functional monomer to develop a fluorescence sensor to detect lactic acid.

**Fig. 4:**
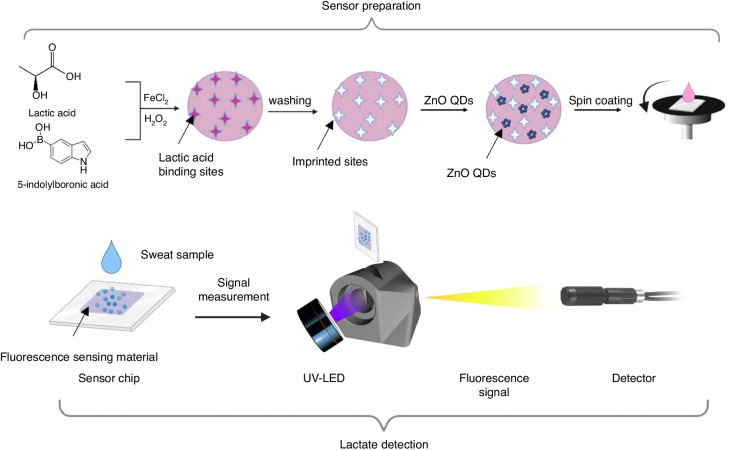
Sensor preparation and detection setup. Schematic representation of sensor preparation and lactate detection processes. The photographs of the detection device, sensor, UV-LED, and the sensing materials under UV illumination are illustrated in Fig. S8

To adapt the synthesis process of MIP@PIn-BAc for ZnO QDs and sensor chip formation, modifications were made to the reported method^38,39^. As schematically represented in Fig. 4, the non-fluorescence MIP@PIn-BAc was formed first, and then ZnO QDs were attached to obtain MIP@PIn-BAc/ZnO fluorescence sensors. We used the two-step fluorescence sensor formation approach to protect ZnO QDs against alkaline and oxidative polymerization conditions. This has also been shown to reduce the emission damage caused by the repeated washing process^56^.

#### Studying the effect of solvent

ZnO QDs demonstrate a higher solubility and fluorescence intensity when dissolved in ethanol, while MIPs show better target recognizability when dissolved in DI water. Therefore, the solvent used to link QDs to the MIPs should be optimized to balance between the fluorescence intensity and the target recognition ability. To realize that, the attachment process was performed in DI water, DI water–ethanol mixture, and pure ethanol to evaluate the effect of solvent on the sensor performance. MIP@PIn-BAc/ZnO prepared with ethanol showed the highest fluorescence intensity ( ~ 70,000 a.u.) because of the compatibility between ethanol and ZnO QDs. A fluorescence intensity around 20,000 a.u. and 10,000 a.u. were detected for sensors prepared with DI water–ethanol mixture and DI water, respectively, which indicated that the sensor’s initial fluorescence intensity decreased with the addition of water (Fig. S5A). The difference in the initial fluorescence intensities indicated the different solvent relaxation of ZnO in ethanol and DI water, where DI water caused quenching of the QDs^57^. These sensors were then treated with the blank solution and 4 mmol L^-1^ lactic acid to evaluate the net sensor response to lactic acid (Fig. S5B). Despite the high response of the sensor prepared with ethanol (*F*_0_/*F* = 2.49), the net response (response to lactate minus response to blank) was only 0.14. For the sensor prepared with pure DI water, although the response to the blank solution was low (*F*_0_/*F* = 1.11), the comparably low response to lactic acid (*F*_0_/*F* = 1.18) resulted in a net response of around 0.07. The highest net response was realized by the sensor prepared with the DI water–ethanol mixture, which had a net response of 0.44. This led to the realization that adding DI water during the sensor preparation reduces the blank response by buffering the quenching effect of water; to use a familiar analogy, giving a “vaccine” to the QDs to prepare the sensor for the analytical evaluation. Therefore, the ZnO QDs sensor treated with “vaccination” would be the optimum candidate for obtaining a high sensor sensitivity.

#### Evaluation of sensor performance for lactic acid

**Fig. 5:**
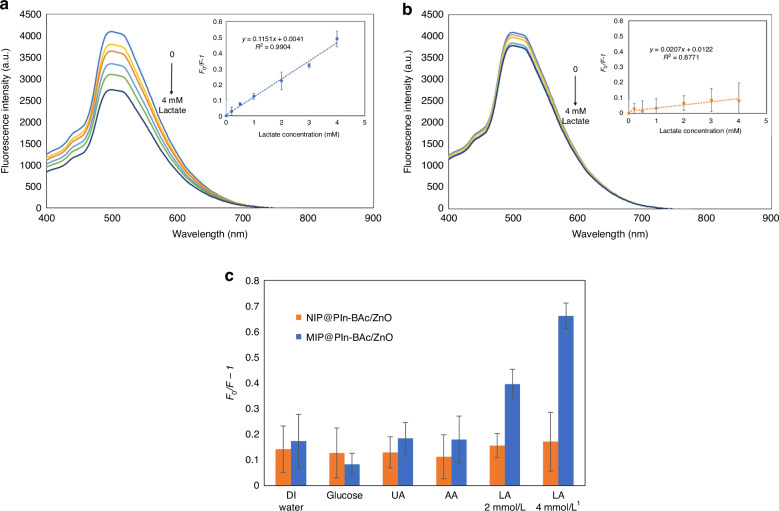
The evaluation of imprinted and non-imprinted PIn-BAc/ZnO sensor sensitivity and selectivity. The response of the MIP@PIn-BAc/ZnO (**a**) and NIP@PIn-BAc/ZnO (**b**) sensors to increasing concentrations of lactic acid in DI water represented with fluorescence spectra and Stern–Volmer plot (inset). NIP and MIP @PIn-BAc/ZnO sensor response to glucose, UA, and AA with the reference response to DI water and lactic acid (**c**)

The MIP@PIn-BAc/ZnO solid sensor prepared with the solvent mixture was evaluated by analyzing its response to various concentrations of lactic acid. The response of the NIP@PIn-BAc/ZnO sensor was also analyzed and compared with the MIP@PIn-BAc/ZnO sensor to assess the effect of imprinted sites. For 0–4 mmol L^-1^ of lactic acid, the fluorescence intensity of the MIP@PIn-BAc/ZnO sensor showed a gradual and consistent decrease with increasing concentrations of lactic acid (Fig. 5a). According to the Stern–Volmer plot, the sensor maintained a linear response curve for 0–4 mmol L^-1^ of lactic acid with a sensor sensitivity of 0.12 and a correlation coefficient of 0.99 (inset of Fig. 5a).

The NIP@PIn-BAc/ZnO fluorescence sensor was not able to show distinguishable changes in the fluorescence intensity to the increasing concentration of lactic acid, as shown clearly in the Stern–Volmer plot in the inset of Fig. 5b; the sensitivity of the sensor was calculated as 0.0207 with a correlation coefficient of 0.88. The imprinting factor, which is the ratio of imprinted and nonimprinted sensors’ sensitivity (Eq. 2), was 5.5. This difference in the sensor performance was due to the imprinted sites embedded in MIP@PIn-BAc/ZnO. The presence of imprinted sites facilitated the binding of lactic acid to the sensor and enabled the recognition of the target molecule in different concentrations, therefore allowing the sensor to exhibit consistent and concentration-specific quenching. In contrast, lactic acid can only bind to the surface boronic acid groups of NIP@PIn-BAc/ZnO and quench the QDs near the surface. When the surface binding sites were saturated, despite the addition of lactic acid in a higher concentration, the response remained at a similar level.

2 $${Imprinting\; factor}={MIP\; sensor\; sensitivity}/{NIP\; sensor\; sensitivity}$$

To determine the effect of interfering molecules, the fluorescence response of the MIP@PIn-BAc/ZnO and NIP@PIn-BAc/ZnO sensors to the common interference molecules in sweat, such as glucose, UA, and AA, was evaluated (Fig. 5b). These interfering molecules can potentially bind to the boronic acid group of the polymer or the amino-functionalized QDs to generate a false-positive sensor response. For the MIP@PIn-BAc/ZnO sensor, the highest quenching was shown by lactic acid, while the response to other molecules was equally low and similar to that of DI water. Therefore, it could be concluded that the response shown to the interference molecules by the MIP@PIn-BAc/ZnO sensor was mainly because of the sensor response to DI water, and that the response induced by the interfering molecules was negligible.

In comparison, the responses of NIP@PIn-BAc/ZnO sensor to the interfering molecule and the blank sample were not differentiable from those of lactic acid. As a result, it can be concluded that the NIP-based sensor was not capable of either detecting or recognizing the target molecule. The performance of the MIP@PIn-BAc/ZnO and NIP@PIn-BAc/ZnO sensors indicated that the recognition of the target molecule by the sensors was due to the molecularly imprinted sites. During the polymer formation, the *cis*-diol functional group of lactic acid was bound to the boronic acid group of the monomer 5-indolylboronic acid, so the specificity of the MIP@PIn-BAc/ZnO sensor was determined by the shape, size, and functional group of the target molecule. All these criteria need to match the imprinted sites to achieve a high sensor response. Therefore, even though both glucose and AA contain more than one *cis*-diol group, the differences in other aspects prevented these molecules from binding with the boronic acid groups on the imprinted sites to induce a high sensor response. For the NIP@PIn-BAc/ZnO sensor, molecules bonded to the functional groups on the polymer surface, which does not display selectivity; therefore, the NIP@PIn-BAc/ZnO sensor could not differentiate lactic acid.

#### Adaptation of sensor for saline condition

The successful measurement of lactic acid in DI water with high sensitivity and selectivity using the MIP@PIn-BAc/ZnO sensor encouraged the evaluation of the sensor in an environment similar to that of sweat. In anaerobic glycolysis, lactate, which is the anion form of lactic acid, is produced by the Cori cycle. PBS is a water-based salt solution commonly used in biological research to represent sweat. Therefore, sample solutions were prepared using PBS to mimic the ion-rich saline content of sweat, where lactic acid loses a proton to form lactate. When the MIP@PIn-BAc/ZnO sensor was used to detect lactate in these samples, the sensor’s response to DI water and PBS samples was significantly different (Fig. S6); an enhancement in the fluorescence intensity was detected for PBS in contrast to the quenching shown in the DI water samples. In addition, the response of the sensor to lactate in PBS did not act in accordance with the Stern–Volmer relation, which indicated that the sensor cannot detect lactate in a saline environment.

Previously, it was observed that incorporating DI water during the composite formation adjusted the sensing material to the conditions of the analysis (lactic acid in DI water) by minimizing the blank response. This process of treating the sensing material with the analysis solution was analogized to providing a ‘vaccine’ to the QDs, preparing the sensor for analytical evaluation, hence the ‘vaccination’ theory. Therefore, when transitioning the sensing condition from DI water to PBS, it was hypothesized that PBS would serve as the ‘vaccine’ or solution to acclimate the sensing material to the new environment and mitigate the influence of the blank.

**Fig. 6:**
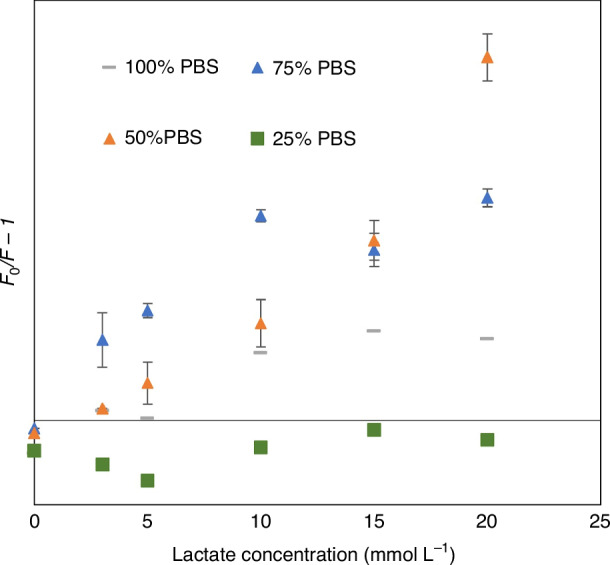
Evaluation of sensor prepared with different PBS “vaccine”. Response of sensors prepared by adding 25%, 50%, 75%, and 100% PBS during the synthesis

Similar to DI water, PBS also affects the fluorescence intensity of the ZnO QDs, so optimization was needed on the amount of PBS to prepare the sensor for the saline environment while preserving its fluorescence intensity. Figure 6 shows the response of sensors prepared with different amounts of PBS. When 25% PBS was added during composite formation, there was still no traceable pattern on the fluorescence quenching. When the amount of PBS was increased to 50%, the zero point (the response to PBS) was negative, but the quenching effect started to take place with the addition of lactate. The quenching of the sensor also increased with target concentration which resulted in a linear response curve for 0–20 mmol L^-1^ of lactate. When the PBS was increased to 75%, the sensor showed a quenching to lactate but the linearity was lower than the sensor prepared with 50% PBS. Finally, when the amount of PBS was increased to 100%, quenching could still be observed, but the degree of quenching was much lower than the sensor prepared with 50% and 75% PBS. This is because there were fewer active QDs left in the sensor when ethanol was eliminated from the synthesis process.

Overall, the “vaccination” was proven to be useful for adapting ZnO QDs to a saline environment; and the amount of PBS added as the vaccine is vital for the sensor performance. This additional step ensured that the chemical environment of the sensing material was more aligned with the analysis conditions. By pre-exposing the sensing material to the blank solution, which also induced quenching, reduced the impact of the blank during the actual sensing process. With this finding, ZnO QDs can potentially be applied to various sensing conditions and can be a “green” replacement for the commonly used Cd-based QDs.

#### Evaluation of sensor performance for lactate

**Fig. 7:**
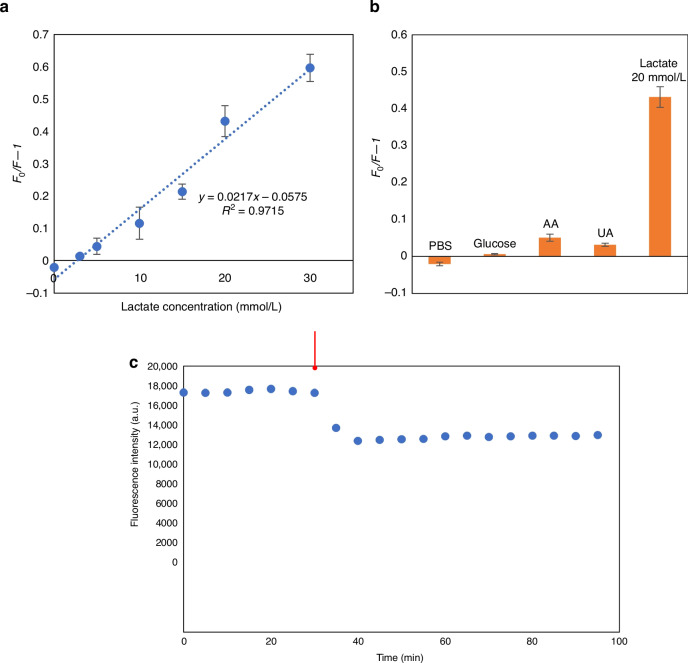
Vaccinated MIP@PIn-BAc/ZnO sensor response in terms of sensitivity, selectivity and stability. The response of the MIP@PIn-BAc/ZnO sensor prepared with 50% PBS to lactate in PBS (**a**) and the same sensor’s response to glucose, UA, and AA with the reference response to PBS and lactate (**b**). The sensor response before (t = 0–30) and after the addition of the target molecule (t = 30–60) measured with ~5 mins intervals, the arrow represent the time of adding lactate (**c**)

The MIP@PIn-BAc/ZnO sensor prepared with 50% PBS was further evaluated on its sensitivity and selectivity for lactate measurement. By recording the sensor response with a wide range of lactate concentrations, the sensor was proven to be capable of responding to 0–30 mmol L^-1^ of lactate (Fig. 7a), with a sensitivity of 0.0217 and a correlation coefficient of 0.97. The limit of detection, calculated using the International Union of Pure and Applied Chemistry (IUPAC) criteria 3σ/S was 3.38 mmol L^-1^, where σ is the standard deviation of the blank solution and S is the slope of the calibration curve^49^. The selectivity of the sensor was evaluated by recording the response to 0.3 mmol L^-1^ glucose, 0.03 mmol L^-1^ AA and 0.05 mmol L^-1^ UA, which are the concentrations of these molecules commonly found in sweat. As shown in Fig. 7b, the sensor’s response to 20 mmol L^-1^ of lactate, which is the lactate concentration in sweat at rest^58^ was much higher than the response to the interfering molecules, thus proving a high sensor selectivity.

The sensing response to lactate in PBS indicates the capability of the developed sensor to measure lactate concentration in sweat with high sensitivity and selectivity. Compared with the sensor’s response to lactic acid in DI water, the sensor showed a wider linear range for lactate in PBS, which is beneficial for capturing large changes in lactate concentration during exercise. This is also the first time ZnO QDs have been successfully applied to detect the target molecule in a saline environment, to the best of our knowledge.

To evaluate the sensor’s stability and repeatability, its fluorescence intensity was measured before and after exposure to the lactate samples in ~5-minute intervals for 100 minutes. As shown in Fig. 7c, the fluorescence intensity of the sensor was measured for 30 minutes before adding lactate, maintaining an average fluorescence intensity of 18,000 a.u. throughout the testing interval, indicating stable fluorescence of the sensor chip. After the addition of lactate at t = 30 mins, the fluorescence plateaued at approximately 14,500 a.u. within about 10 minutes of reaction time. The relative standard deviation in the plateaued region was 0.02, indicating the high stability and repeatability of the results within the system.

To assess the MIP@PIn-BAc/ZnO sensor’s applicability for long-term use, the response of the sensor was measured after a month of storage. The results demonstrated that the sensor maintained 60% of its original response to lactate after a month (Table 1). Optimizing the sensor to attain good stability was beyond the scope of this study, whose focus was evaluating the possibility of lactate measurement with fluorescence sensor chips. However, long-term storability can be attained by further improving the sensing material. The adsorbed ions during the adaptation step, which might have impaired QDs over time, most likely resulted in a lower sensor response. Applying a protective layer on the QDs may help to achieve a more stable structure and increase the storability of the QDs. In addition, the passivation step may be eliminated with the core–shell structure since the QDs are sheltered from the saline environment. Therefore, long-term stability can be obtained with some modification of the developed sensing system.

**Table 1:** Response of the sensor when newly prepared and after one month of storage

	Response to blank	Response to 10 mM Lactate	Response to 20 mM Lactate	Response to 30 mM Lactate	Sensitivity (considering 4 points)
New Sensor	0	0.11	0.43	0.60	0.021
After one month	0	0.06	0.24	0.36	0.013

In this study, the lactate sensor was tested in PBS buffer, commonly used in biological research to simulate sweat. Other buffers mimicking sweat composition could also be employed to evaluate sensor response. However, the sensing performance may vary due to two primary reasons. First, different buffers affect the fluorescence intensity of the QDs due to solvent relaxation, with varying quenching effects from specific anions^59,60^. Thus, using a different buffer is likely to alter the sensing response. Additionally, the pH of the buffer solution can influence the dissociation state of lactic acid^60,61^. Given that the imprinted sites of MIP@PIn-BAc/ZnO were formed in an alkaline environment, they preferentially interact with the lactate form, impacting sensor response based on the amount of lactate present and the buffer pH. Nevertheless, we anticipate that lactate will quench the emission of the sensing material and that the ‘vaccination’ method will better prepare the material for the new sensing environment, irrespective of the buffer type. The detailed examination of buffer effects falls beyond the scope of this study; we encourage further investigation in this area.

#### Performance comparison of sensors formed with different monomers

When compared to MIP@PIn-BAc/ZnO, ZnO@APTES-MIP exhibited a 32% higher sensitivity but significantly lower selectivity. This sensitivity difference could be attributed to variations in the preparation process. In the case of MIP@PIn-BAc/ZnO, the ZnO QDs underwent treatment with DI water, intentionally utilizing the quenching effect of water to enhance the net response. Conversely, in ZnO@APTES-MIP, such treatment was unnecessary because the QDs were embedded within the polymer network, shielding them from the quenching effect of the blank solution. Therefore, the deliberate quenching of QDs in the MIP@PIn-BAc/ZnO composite likely contributed to its reduced sensitivity compared to ZnO@APTES-MIP, despite the same initial quantity of QDs (0.7 g).

Differences in the selectivity of ZnO@APTES-MIP and MIP@PIn-BAc/ZnO sensors revealed the effect of monomer on sensor performance. The MIP@PIn-BAc/ZnO sensor could recognize lactic acid (showed a negligible response to the interfering molecules), whereas the ZnO@APTES-MIP sensor could not recognize lactic acid (showed a similar response to lactic acid and the interfering molecules). The differences in lactic acid recognition ability were attributed to the differences in the binding affinity of lactic acid with the monomers. It can be interpreted from the sensor response that although both monomers contain functional groups that can bind with the target (Fig. S7), imprinted sites were only formed when the monomer was binding to the *cis*-diol group of lactic acid. The unsuccessful formation of imprinted sites with APTES might be because lactic acid was presented in the deprotonated state during the synthesis process. Rambow, J et al.’s study showed that the fraction of natural lactic acid would decrease to 0% when PH is higher than 6^62^. It was assumed that lactic acid lost a proton to form lactate during the synthesis because of the addition of ammonia and NaOH. Therefore, the anticipated binding between the amino functional group of APTES and the carboxyl functional group of lactic acid could not take place, given that the lactic acid was presented in its salt form lactate. In comparison, higher PH was favorable for the binding between the *cis*-diol group and the boronic acid group, so the imprinted sites were formed successfully with the 5-indolylboronic acid monomer, thus resulting in a higher selectivity.

The variation in selectivity between the sensors using APTES and 5-indolylboronic acid as monomers underscores the importance of selecting monomers based on their affinity for the target template molecule. To establish reliable imprinted sites, these interactions should not be overly influenced by fluctuating physical conditions such as temperature and pH.

#### Performance comparisons of the developed sensor and other sensing techniques

Table 2 presents a comparative analysis of the sensor developed in this study with other relevant sensors, including a molecularly imprinted capacitive sensor, lactate-specific molecularly imprinted polymer, enzyme-based electrochemical microbiosensors, and an electrochemical sensor. The Molecularly imprinted capacitive sensor exhibited a detection range similar to that developed in this study, albeit with a lower detection limit. The Molecularly imprinted Ag nanowires-based electrochemical sensor displayed a wider detection range and a significantly lower detection limit than the fluorescence sensor developed in this study. However, our sensor demonstrated notably higher sensitivity than the other sensors, owing to its ability to yield significant responses even with minimal concentration changes—an advantage of using fluorescence sensors.

**Table 2:** Performance comparisons of various molecularly imprinted sensors

	Detection Range (mM)	Limit of Detection (mM)	Sensitivity	Linearity	Reference #
Molecularly imprinted capacitive sensor	1–25	0.1	2.00E-05	0.9908	^63^
Molecularly imprinted Ag nanowires-based electrochemical sensor	0.001–100	0.00022	4.50E-06	0.995	^13^
Lactate-specific molecularly imprinted polymer	0.1–1.7	0.162	unknown	0.99	^64^
Enzyme-based electrochemical microbiosensors	0-1	6.1×10^-3^	unknown	unknown	^65^
Solid-phase molecularly imprinted fluorescence sensor	0–30	3.38	0.0217	0.9715	This study

The lactate-specific molecularly imprinted polymer achieves a lower detection limit compared to our study. However, it relies on UV-VIS spectroscopy for signal reading and operates in the liquid phase. In contrast, our study introduces a sensor chip with a portable light detector, addressing issues related to detection location and the need for expensive equipment. Enzyme-based electrochemical microbiosensors offer a remarkably low detection limit and specific detection for lactate in blood serum. However, these sensors often suffer from poor stability due to enzyme deactivation and degradation in response to pH and temperature fluctuations. In contrast, the MIP-based sensor developed in this study is expected to be more resilient under various environmental variations compared to enzyme-based sensors. Additionally, the detection and preparation processes used for the developed sensor are more straightforward than those of enzyme-based sensors.

In sum, our sensor showed promising results in detecting lactate in PBS—an approach that is commonly used in biological research involving sweat analysis to mimic the sweat environment. To further assess the sensor’s applicability in sweat analysis, the developed sensor needs to be evaluated with actual sweat samples for lactate measurement, and these results should be statistically analyzed and compared with the results obtained using the established methods to measure the sensor’s accuracy.

## Conclusion

The first account of a solid fluorescence sensor that combined the high sensitivity of ZnO QDs and the high selectivity of MIPs showed superior performance in detecting lactic acid in DI water and lactate in PBS. Especially, the large linear range (0–30 mmol L^-1^) and the high selectivity of the sensor to the lactate in PBS proved the possibility of using this cost-efficient fluorescence sensor to detect lactate in actual sweat samples.

The novel aspects of this study include pioneering the utilization of ZnO QDs in biomarker detection and developing adaptable protocols for integrating ZnO QDs into diverse sensing environments. A simple ‘vaccination’ method was proven effective for preparing ZnO QDs for sensing applications. Additionally, the study introduces an innovative approach to forming a sensing chip by employing a physical immobilization technique. This technique facilitates the seamless transition from liquid QDs-based sensing to the solid phase, enabling the substitution of fluorescence spectroscopy with a portable light detector.

The innovations presented in this work take the synthetic biosensor a step closer to a commercial product. Also, on account of the synthetic nature of the MIP receptor and the wide-range applicability of the fluorescence materials, the developed sensing materials can be easily modified to detect other biomarkers to offer comprehensive body function monitoring.

## Supplementary information

Supplemental material
