# Supplementary material for: Molecularly imprinted fluorescence sensor chip for lactate measurement
Source: Microsyst Nanoeng. 2024 Nov 25;10:175. doi: 10.1038/s41378-024-00803-4 (PMC11586410; doi:10.1038/s41378-024-00803-4)
Supplement: Supplementary file 1 — Supplemental material [file 41378_2024_803_MOESM1_ESM.docx]

**Supplementary Material**

**Molecularly Imprinted Fluorescence Sensor Chip for Lactate Measurement**

Muersha Wusiman, Fariborz Taghipour *

Chemical and Biological Engineering, University of British Columbia, Vancouver, V6T 1Z3, Canada.

* Corresponding Author

E-mail: [fariborz.taghipour@ubc.ca](mailto:fariborz.taghipour@ubc.ca)

A


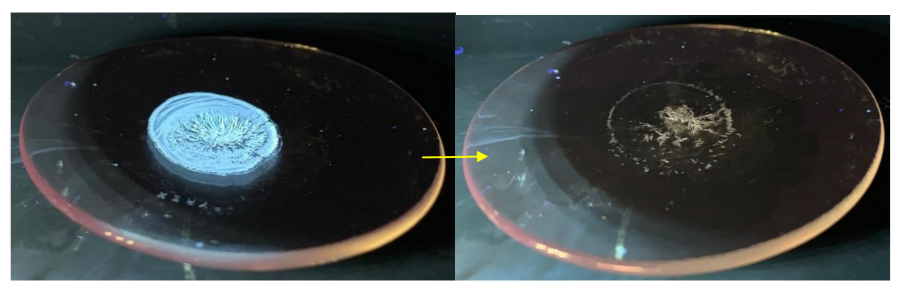

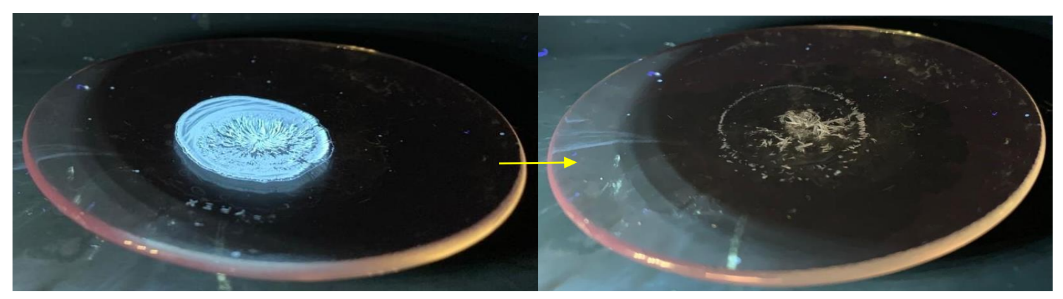


UV excitation

Fig. S1. A. Absorbance and the emission spectra of ZnO QD recorded with UV-VIS spectroscopy and fluorescence spectroscopy respectively (please print with color). B. The emission of ZnO QDs under natural light and under UV light.

**Fig. S2.** The response of the ZnO QDs for lactate in increasing concentration.


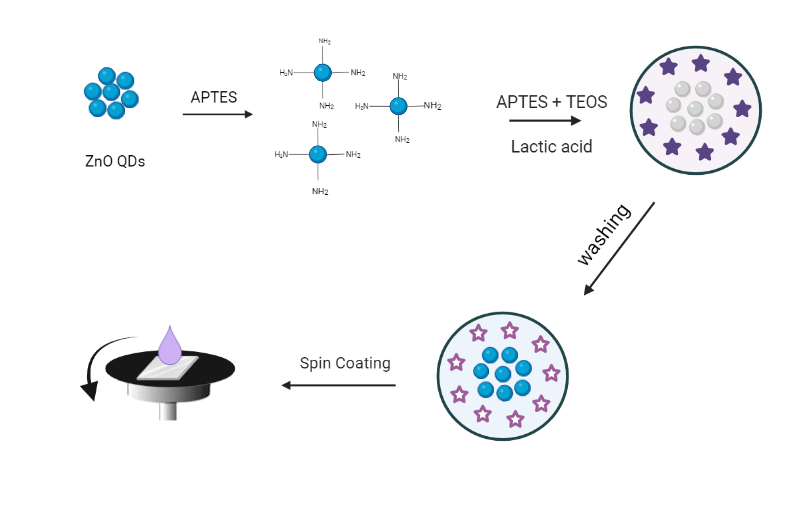


Fig. S3. Schematic representation of lactic acid MIP synthesis procedure with APTES monomer. First, ZnO QDs were functionalized with APTES to increase the stability, then an imprinted polymer layer was formed on the surface of the QDs. After removing the template molecules from the polymer, the sensing material was coated on quartz to form MIFS chips (please print with color).


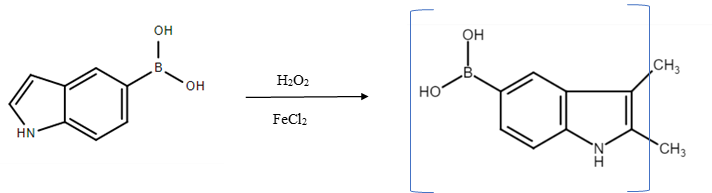


A

B

Fig. S4. A. Indolyl group polymerization. B. binding between boronic acid and lactic acid.

A

Fig. S5. A. Initial fluorescence intensity of the sensors prepared with DI water, ethanol and the mixture of ethanol and DI water and B. the response of these sensors to the blank solution (orange bar) and 4 mM of lactic acid (blue bars) (please print with color).

Fig. S6. The response of MIP@PIn-BAc/ZnO sensor to lactic acid in DI water (Lac-DI) and lactate in PBS (Lac-PBS) (please print with color).


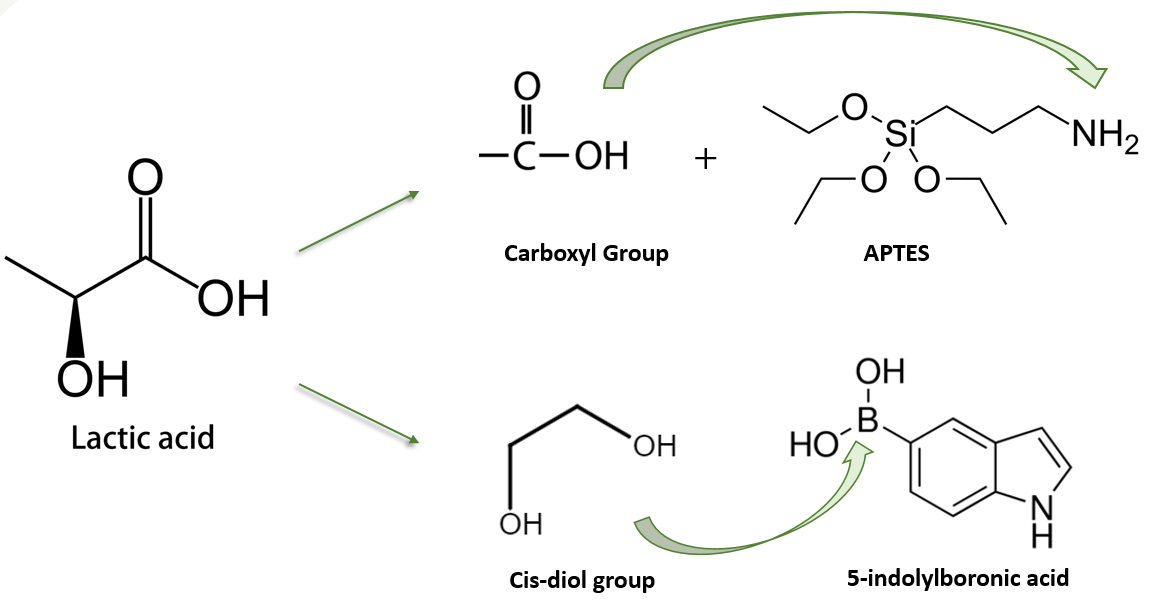


Fig. S7. Representation of the two functional groups of lactic acid and the monomers chosen in this study, the arrows show the binding sites between the functional group and the monomer.


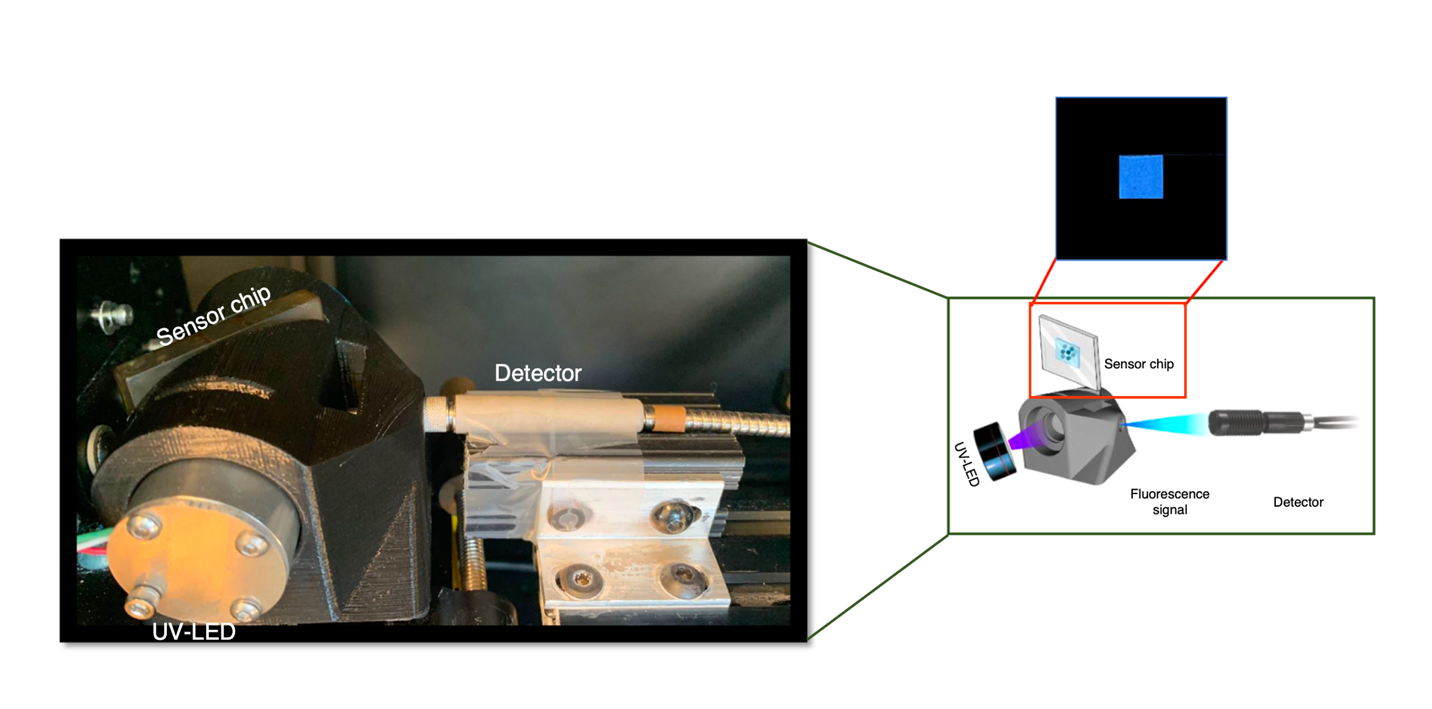


**Fig. S8.** The photographs of the detection device, sensor, UV-LED and the sensing materials under UV illumination.
